# Inhibitory effect of plant flavonoid cyanidin on oral microbial biofilm

**DOI:** 10.1128/spectrum.02848-24

**Published:** 2025-04-30

**Authors:** Lucille Rudin, Julien Kneubühler, Badri Nath Dubey, Sabahuddin Ahmad, Michael M. Bornstein, Viktoriya Shyp

**Affiliations:** 1 Department Research, University Center for Dental Medicine Basel UZB, University of Basel 27209, Basel, Switzerland; 2 CSSB Centre for Structural Systems Biology, Deutsches Elektronen-Synchrotron DESY 28332, Hamburg, Germany; 3 Institute of Bio- and Geosciences, IBG-1: Biotechnology, Forschungszentrum Jülich GmbH 234656 , Jülich, North Rhine-Westphalia, Germany; 4 Department of Oral Health & Medicine, University Center for Dental Medicine Basel UZB, University of Basel 27209, Basel, Switzerland; The Ohio State University College of Dentistry, Columbus, Ohio, USA

**Keywords:** biofilms, *Streptococcus mutans*, flavonoids, dental plaque

## Abstract

As primary colonizers of the tooth surface, oral streptococci play a crucial role in dental caries development. Numerous natural compounds, including flavonoids, are emerging as promising agents for inhibiting dental biofilm formation without compromising bacterial viability, underscoring their potential in non-bactericidal antibiofilm strategies. This study investigated the effects and mechanism of action of the unmodified plant flavonoid cyanidin on the growth and sucrose-dependent biofilm formation of oral streptococci, with a particular focus on the cariogenic pathogen *Streptococcus mutans*. At concentrations above 100 µg/mL, cyanidin significantly inhibited biofilm formation by *S. mutans* without impacting bacterial viability. The flavonoid reduced the biomass of surface-associated bacteria and exopolysaccharides (EPS), particularly by inhibiting water-insoluble glucan (WIG) production mediated by the glucosyltransferases GtfB and GtfC. While cyanidin did not exhibit a bactericidal effect on early colonizer streptococci, such as *Streptococcus sanguinis*, *Streptococcus gordonii*, *Streptococcus oralis*, and *Streptococcus mitis*, it showed a significant inhibitory effect on bacterial acidogenicity and mixed-species streptococcal biofilms in the presence of *S. mutans*. Remarkably, cyanidin gradually reduced the proportion of *S. mutans* in the mixed biofilm, suggesting a selective impact that may promote a more commensal-dominant community by disrupting *S. mutans* glucan production and biofilm competitiveness.

**IMPORTANCE:**

The identification of compounds with potent antibiofilm effects that do not compromise bacterial viability presents a promising strategy for oral health management. By preventing biofilm formation and keeping bacteria in a planktonic state, such agents could enhance bacterial susceptibility to targeted therapies, including probiotics or phage-based treatments. Cyanidin, which exhibits strong antibiofilm activity against oral streptococcal biofilms, reduces bacterial acidogenicity and may promote a more commensal-dominant biofilm *in vitro*, potentially hindering the maturation of cariogenic biofilms.

## OBSERVATION

**Fig 1:**
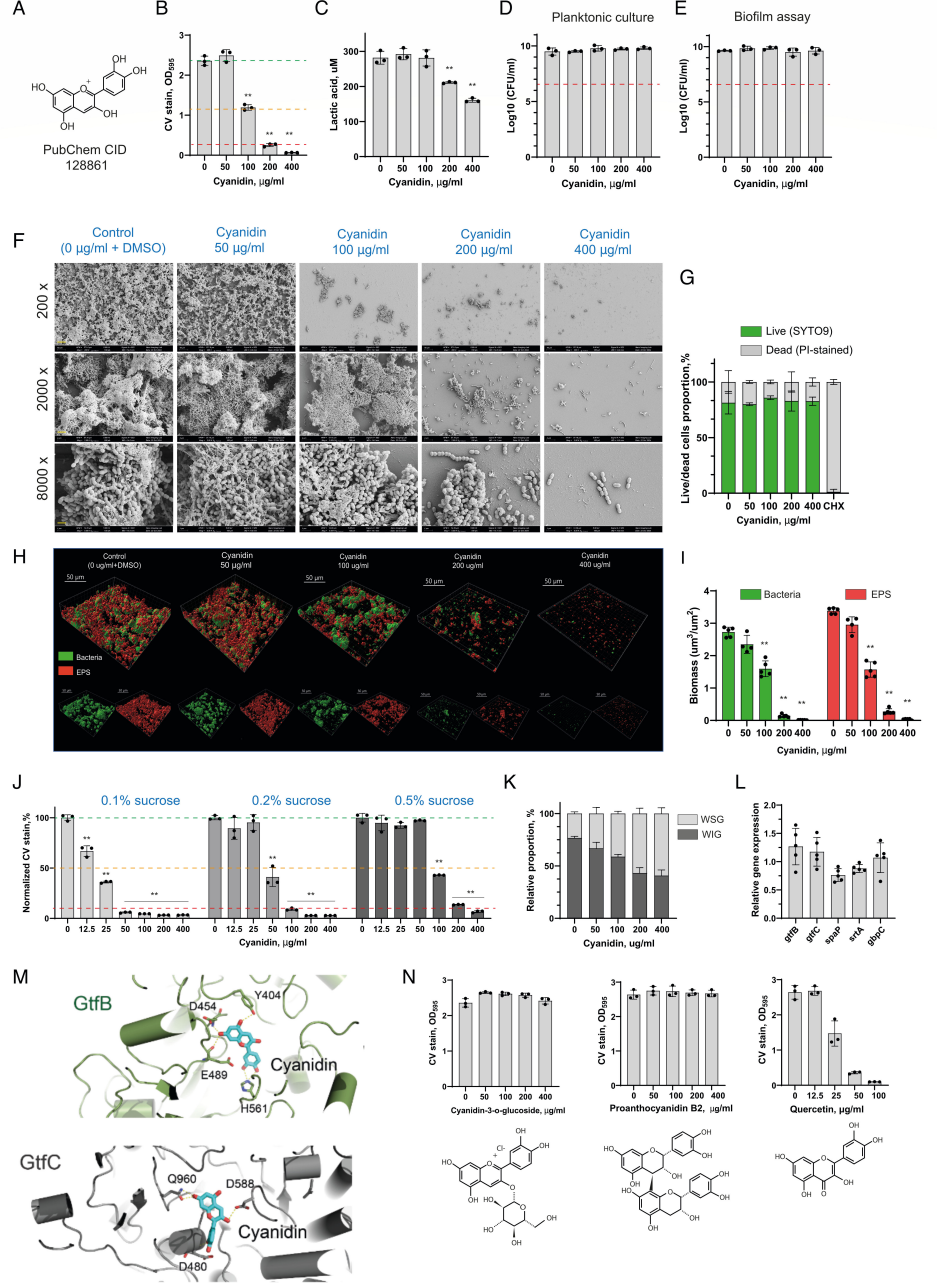
Antibiofilm activity of cyanidin against *S. mutans*. (**A**) Chemical structure of cyanidin with PubChem ID indicated. (**B**) The effect of cyanidin on 24 h biofilms of *S. mutans* formed on a 96-well plate in brain heart infusion broth supplemented with 1% sucrose (BHIS). Biofilms were quantified by crystal violet staining and measuring absorbance at 595 nm. Green, orange, and red dashed lines indicate 0%, 50%, and 90% of biofilm inhibition, respectively. (**C**) Production of lactic acid by *S. mutans* treated with 50, 100, 200, and 400 µg/mL of cyanidin. (**D and E**) The effect of cyanidin on *S. mutans* cell viability growing for 24 h in planktonic culture (**D**) or collected all together after the biofilm assay (**E**). After the incubation, cell suspensions were serially diluted and plated for CFU enumeration. Red dashed lines indicate a 3-log_10_ reduction (99.9% reduction in CFU/mL) considered bactericidal. (**F**) Representative image from scanning electron microscopy (SEM) analysis of *S. mutans* biofilm formed on borosilicate glass disks submerged in a 96-well plate for biofilm assay. Disks were observed at magnifications of 200×, 2,000×, and 8,000× in three arbitrarily selected locations. The yellow scale bar represents 40, 4, and 1 µm for respective magnifications. (**G**) The proportion of live/dead cells was calculated from the cell biomasses reconstructed from CLSM images. “Live” cell fraction corresponds to the SYTO9-stained cells, while “dead” corresponds to the PI-stained cells. Chlorhexidine, 0.02%, was used as a bactericidal control. (**H**) Representative images of the double-labeled 24 h biofilms treated with cyanidin. Bacterial cells are shown in green, and exopolysaccharide (EPS) is in red. Three-dimensional reconstructions based on fluorescent intensity values were performed with Imaris 9.0.0. (**I**) Bacterial and EPS biomasses as calculated by Imaris per observational area after the three-dimensional reconstructions. (**J**) The effect of cyanidin on 24 h biofilms of *S. mutans* formed in the presence of 0.1%, 0.2%, and 0.5% sucrose. For each condition, CV staining values have been normalized to the non-treated control. Green, orange, and red dashed lines indicate 0%, 50%, and 90% of biofilm inhibition, respectively. (**K**) The effect of cyanidin on water-soluble and water-insoluble glucans produced by *S. mutans* cells in a 24 h biofilm assay, as measured by the anthrone-sulfuric method. (**L**) The effect of 200 µg/mL cyanidin on the biofilm-associated gene expression levels in a 24 h biofilm. The relative expression levels were quantified by real-time PCR with 16S rRNA as an internal control. (**M**) The best docked interaction of cyanidin with the active sites of GtfB (green) and GtfC (gray) of *S. mutans*. The cyanidin molecule is shown in cyan, and hydrogen bonds with displayed amino acids are shown as dashed yellow lines. (**N**) The effect of cyanidin-3-*O*-glucoside, proanthocyanidin B2, and quercetin on 24 h biofilms of *S. mutans* formed on a 96-well plate in brain heart infusion broth supplemented with 1% sucrose (BHIS). In all panels, bacterial culture supplemented with 5% dimethyl sulfoxide (DMSO) was used as a control. Bars represent the mean of at least three biological replicates. Error bars show standard deviation. **P < 0.0001.

Bacterial biofilms are multicellular, surface-associated communities encased in a self-produced extracellular matrix, exhibiting greater resistance to antibiotics and environmental stresses compared to their planktonic counterparts (1). The human oral cavity harbors diverse microbial communities that live in biofilms and are recognized as a virulence factor in many oral infectious diseases, including dental caries and periodontitis (2). Dental biofilm development starts with non-specific attachment of early colonizers, mainly oral streptococci, to the salivary pellicle covering the dental surface and is followed by the accumulation of bacteria within a more complex and structured microbial community (3). As a result of increased dietary sugar intake and excess acid production by oral bacteria, a symbiotic oral microbial community may shift to a dysbiotic community with the prevalence of pathogenic bacteria. Dental caries develops as a consequence of dietary sugar-driven microbial growth and carbohydrate metabolism that leads to localized acidification and tooth demineralization. Among highly acidic and acid-tolerant species, *Streptococcus mutans* is often recognized as a causative agent of dental caries. It gained a significant evolutionary advantage for persisting and surface colonization via the ability to metabolize a large variety of carbohydrates, including one of the most cariogenic ones, sucrose (4, 5). At the same time, dental health is generally associated with a higher proportion of commensal early colonizers such as *Streptococcus sanguinis* and *Streptococcus parasanguinis*, *Streptococcus gordonii*, *Streptococcus mitis*, or *Streptococcus oralis* (6). Hence, effective dental therapies should target virulence factors and pathogenic traits of oral bacteria selectively, aiming to preserve beneficial commensal microbes essential for maintaining oral health. Numerous natural compounds, including flavonoids, are emerging as promising agents due to their ability to inhibit dental biofilm formation without adversely affecting bacterial viability (7–9). Recently, cyanidin (Fig. 1A), an anthocyanidin flavonoid isolated from various plants, has been identified as a non-bactericidal effective inhibitor of biofilm formation in methicillin-resistant *Staphylococcus aureus* (10) and the opportunistic pathogen *Klebsiella pneumoniae* (11). In *S. mutans*, glycosylated anthocyanidins from cranberry extract, such as cyanidin or peonidin, have shown no significant effect against bacterial biofilm formation and acidogenicity (12). However, this lack of effect may be attributed to their conjugation with sugar moieties, while the effect of unmodified, pure components remains unknown. In this study, the effect of cyanidin aglycone on *S. mutans* growth, acidogenicity, and biofilm formation, as well as its influence on the multispecies community of *S. mutans* with oral commensal streptococci, was investigated to expand our understanding of plant flavonoids as potential antibiofilm compounds relevant for dental medicine.

Using the microbroth dilution assay, the effect of cyanidin on sucrose-dependent biofilm formation of *S. mutans* was tested. At a concentration of 100 µg/mL, cyanidin reduced around 50% of mature biofilm formed after 24 h of incubation, while higher concentrations effectively inhibited above 90% of biofilm (Fig. 1B). Cyanidin also exhibited a suppressive effect on *S. mutans* acidogenicity. At its higher biofilm-inhibitory concentrations, it notably reduced the production of lactic acid—predominant acidogenic metabolite generated by *S. mutans* during sucrose consumption (Fig. 1C). The viability of *S. mutans* cells growing either in planktonic culture without sucrose or collected after biofilm assay revealed no antibacterial effect of cyanidin at its antibiofilm concentrations (Fig. 1D and E). Similarly, cells solely associated with the biofilm after cyanidin treatment retained a similar proportion of membrane-compromised (propidium iodide-stained) cells as a non-treated control (Fig. 1G). Microscopy analysis of *S. mutans* biofilm architecture and composition confirmed a dose-dependent inhibitory effect of cyanidin on surface-associated cells as well as on extracellular polysaccharide (EPS) biomasses (Fig. 1F, H, and I).

Our findings also revealed that the antibiofilm efficacy of cyanidin is influenced by sucrose concentration, with higher sucrose levels requiring increased cyanidin concentrations for effective inhibition. Thus, in the presence of 0.1% sucrose, cyanidin inhibited over 90% of biofilm formation at a concentration of 50 µg/mL. With 0.2% sucrose, 100 µg/mL of cyanidin was needed to achieve similar inhibition. At 0.5% sucrose, 200 µg/mL of cyanidin was required to inhibit over 90% of biofilm formation, a result comparable to that observed with 1% sucrose used in the biofilm formation assay in this study (Fig. 1J). This suggests that cyanidin may primarily interact with sucrose-mediated biofilm formation, potentially through modulation of glucosyltransferase (GTF) activity or EPS synthesis. *S. mutans* produces several types of EPS in its biofilms, with glucans and fructans being the primary types. Glucans are the most significant fraction of EPS synthesized from sucrose by the glucosyltransferase enzymes (GtfB, GtfC, and GtfD) (13). Water-soluble glucans (WSG) produced by GtfD aid in early biofilm formation and nutrient storage. At the same time, water-insoluble glucans (WIG), mainly produced by GtfB and partially by GtfC, enable firm adhesion to the tooth surface, forming a matrix that traps bacteria and acids, increasing the cariogenic potential of the dental biofilm. Quantification of WSG and WIG by the anthrone-sulfuric acid method revealed the selective inhibitory effect of cyanidin on WIG production by *S. mutans* growing in biofilm (Fig. 1K).

Flavonoids have been identified to inhibit GTFs at transcriptional levels (8, 14, 15). As an example, the flavones apigenin and luteolin were found to inhibit *gtfBC* gene expression, thereby reducing glucan production. Interestingly, cyanidin did not significantly affect the expression of the *gtfBC* operon, nor other biofilm-related genes such as *srtA*, *spaP*, and *gbpC*, which encode a transpeptidase, surface adhesin, and glucan-binding protein, respectively (Fig. 1L). This suggests that it could potentially interfere with biofilm-related virulence factors at posttranscriptional levels. To further investigate the potential of cyanidin to inhibit the activity of glucosyltransferases contributing to the WIG synthesis, GtfB and GtfC, molecular docking studies were performed. Figure 1M shows the best-docked pose of cyanidin in the active sites of GtfB and GtfC. Best-docked pose here refers to a conformation in the largest cluster with the least free energy of binding of flavonoid to the respective enzymes. *In silico* docking analysis revealed that cyanidin formed hydrogen bonds with multiple conserved amino acids in the subsite-1 of GtfC, including Asp480, Glu960, and, particularly, Asp588, responsible for the binding and stabilizing of the glucosyl moiety of the sucrose during trans-glycosylation reaction. Similarly, cyanidin interacts with several conserved amino acids in the active site of GtfB, including Glu489, which is involved in Gtf's inhibitor acarbose binding. As observed with other polyphenols that interfere with *S. mutans* virulence enzymes (12, 16–18), further biochemical studies are required to validate the interference of cyanidin with the enzymatic activities of GtfB and GtfC. Additionally, other molecular targets in *S. mutans* could be tested for cyanidin inhibition, such as sortase A (SrtA), a transpeptidase enzyme responsible for anchoring surface adhesins and, therefore, promoting initial bacterial adhesion and subsequent biofilm formation on tooth surfaces. In *Staphylococcus aureus*, cyanidin inhibits the enzymatic activity of SrtA without affecting its expression or the viability of the bacterium (10).

Although the use of natural products and their derivatives has been recognized as a promising approach for inhibiting GTF activity and dental biofilm formation (19), several challenges remain. In particular, natural antibiofilm compounds often require optimization to improve their solubility, specificity, and pharmacokinetic properties. Thus, understanding the structure-function relationships of these compounds is crucial for designing more effective and targeted antibiofilm therapies. Our findings highlight the significance of structural modifications in determining cyanidin bioactivity.

Thus, in line with previous observations regarding the lack of activity of anthocyanidins’ galactoside and arabinoside on *S. mutans* biofilm (12), 3-*O*-glycosylation of cyanidin completely abrogates its antibiofilm activity (Fig. 1N). The addition of a glucose moiety increases the hydrophilicity of the molecule, which likely reduces its ability to interact with bacterial cell walls and biofilm matrices, thereby diminishing its antibiofilm efficacy (20). In contrast, the carbonyl group at the C4 position in the otherwise structurally similar flavonol quercetin increases its hydrophobicity, which may explain its relatively greater antibiofilm activity (Fig. 1N). Additionally, proanthocyanidin B2, a polymerized flavonoid form, lacked detectable antibiofilm activity, suggesting that polymerization may also compromise its ability to inhibit biofilm formation. The pronounced antibiofilm activity of proanthocyanidins reported in previous studies (12, 21) may be attributed to the presence of both monomeric and polymeric forms within the same fraction, where the active monomers contribute to biofilm inhibition. These findings underscore the critical role of structural variations in shaping flavonoid bioactivity, reinforcing the need for detailed structure-function analysis in the development of optimized antibiofilm compounds.

**Fig 2:**
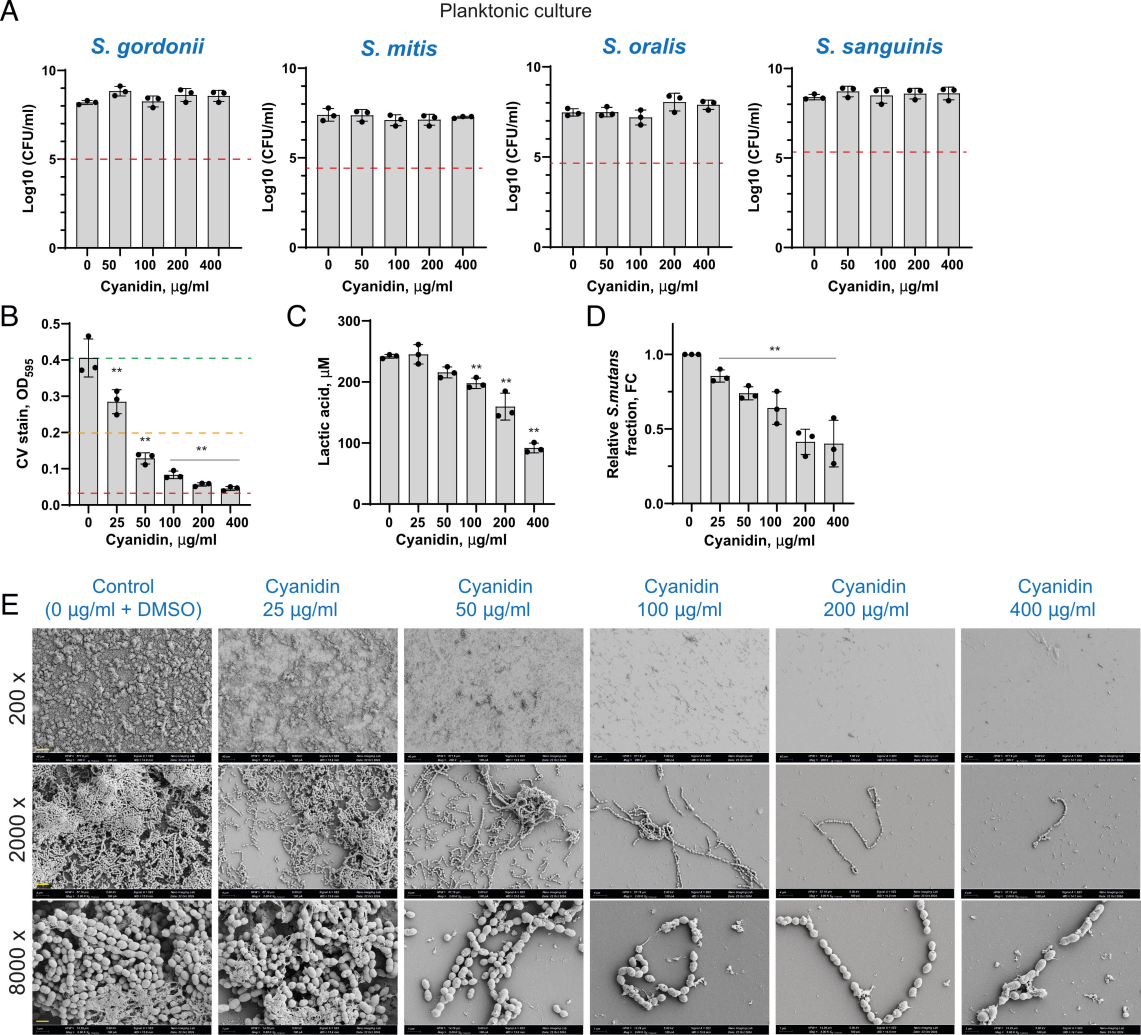
Cyanidin effect on commensal oral streptococci. (**A**) The effect of cyanidin on oral streptococci cell viability growing for 24 h in planktonic culture. After the incubation, cell suspensions were serially diluted and plated for CFU enumeration. Red dashed lines indicate a 3-log_10_ reduction (99.9% reduction in CFU/mL) considered bactericidal. (**B**) The effect of cyanidin on a 24 h mixed streptococcal biofilm (*S. mutans* together with commensal streptococci such as *S. sanguinis*, *S. mitis*, *S. oralis*, and *S. gordonii*) formed on a 96-well plate in brain heart infusion broth supplemented with 1% sucrose (BHIS). Biofilms were quantified by crystal violet staining and measuring absorbance at 595 nm. Green, orange, and red dashed lines indicate 0%, 50%, and 90% of biofilm inhibition, respectively. (**C**) Production of lactic acid by streptococcal mixture treated with 25, 50, 100, 200, and 400 µg/mL of cyanidin. (**D**) The fold change values of the *S. mutans* fraction in treated mixed biofilms relative to untreated controls, calculated using the ΔΔCt method. In all panels, bacterial culture supplemented with 5% DMSO was used as a control. Bars represent the mean of at least three biological replicates. Error bars show standard deviation. **P < 0.0001. (**E**) Representative image from SEM analysis of mixed streptococcal biofilms formed on borosilicate glass disks submerged in a 96-well plate for biofilm assay. Disks were observed at magnifications of 200×, 2,000×, and 8,000× in three arbitrarily selected locations. The yellow scale bar represents 40, 4, and 1 µm for respective magnifications.

While the monospecies biofilm model provides valuable insights into *S. mutans* biofilm characteristics, biofilms in the oral cavity are often composed of multiple species, which can influence bacterial behavior and virulence. To more accurately reflect the complex oral environment, we extended our study to include multispecies biofilms comprising *S. mutans* along with *S. sanguinis*, *S. gordonii*, *S. oralis*, and *S. mitis*. Similar to *S. mutans*, cyanidin did not show bactericidal effect on non-mutans streptococci (Fig. 2A). At the same time, mixed biofilm turned out to be more susceptible to cyanidin treatment. Thus, at a concentration of 25 µg/mL, cyanidin already notably reduced biofilm biomass, while higher concentrations of flavonoid reduced from 50% to 90% of biofilm accumulated after 24 h (Fig. 2B). SEM analysis of the multispecies biofilm supported the dose-dependent inhibitory effect of cyanidin (Fig. 2E). Similar to the monoculture of *S. mutans*, cyanidin reduced the acidogenicity of the streptococcal mixture as measured by lactic acid assay (Fig. 2C). Notably, this reduction was more pronounced in the streptococcal mixture than in the *S. mutans* monoculture (Fig. 1C and 2C), suggesting a potential selective effect of cyanidin on *S. mutans* virulence factors. Furthermore, by disrupting glucan-dependent adhesion and compromising the structural integrity of the biofilm matrix, cyanidin may weaken *S. mutans’* competitiveness within the mixed community, thereby favoring the relative abundance of commensal streptococci. To further investigate whether cyanidin alters the balance between streptococci, we quantified the relative abundance of *S. mutans* within the mixed biofilm following cyanidin treatment using quantitative PCR (qPCR) analysis based on the ΔΔCt method. As shown in Fig. 2D, cyanidin induced a concentration-dependent reduction in the *S. mutans* fraction within the mixed biofilm. To assess total biofilm biomass, the universal bacterial 16S rRNA primers were used, while *S. mutans*-specific primers targeting the *gtfB* gene were employed to determine its relative proportion. However, cyanidin does not completely eliminate *S. mutans* from the biofilm, suggesting that additional factors may influence the microbial shift and prevent complete displacement of cariogenic pathogens within the mixed biofilm. In the mixed biofilm model used in this study, oral streptococcal attachment may rely on both glucan-dependent and glucan-independent mechanisms. Commensal streptococci produce GTFs and glucan-binding proteins (GBPs), which facilitate their aggregation within glucan-rich biofilms. Consequently, the inhibition of *S. mutans* glucan production may also reduce the binding of other bacteria within the biofilm. Additionally, GTFs and GBPs produced by commensal streptococci could serve as potential targets for flavonoid-mediated inhibition, further influencing biofilm composition and structural integrity.

In conclusion, cyanidin demonstrates clear antibiofilm properties against both single-species *S. mutans* and multispecies oral biofilms, significantly reducing biofilm formation and bacterial acidogenicity without compromising microbial viability. Additionally, its ability to inhibit *S. mutans* virulence factors while promoting a shift toward a more commensal-dominated biofilm suggests a potential role in rebalancing the oral microbiome. These findings highlight cyanidin’s potential as a non-bactericidal therapeutic for oral biofilm management, warranting further investigation into its application within targeted, multi-compound treatments for enhanced oral health.

## References

[1] Flemming H-C, Wingender J, Szewzyk U, Steinberg P, Rice SA, Kjelleberg S. 2016. Biofilms: an emergent form of bacterial life. Nat Rev Microbiol 14:563–575. doi:10.1038/nrmicro.2016.94 27510863

[2] Mira A, Simon-Soro A, Curtis MA. 2017. Role of microbial communities in the pathogenesis of periodontal diseases and caries. J Clin Periodontol 44:S23–S38. doi:10.1111/jcpe.12671 28266108

[3] Lemos JA, Palmer SR, Zeng L, Wen ZT, Kajfasz JK, Freires IA, Abranches J, Brady LJ. 2019. The biology of Streptococcus mutans. Microbiol Spectr 7. doi:10.1128/microbiolspec.GPP3-0051-2018 PMC6615571 30657107

[4] Willenborg J, Goethe R. 2016. Metabolic traits of pathogenic streptococci. FEBS Lett 590:3905–3919. doi:10.1002/1873-3468.12317 27442496

[5] Paes Leme AF, Koo H, Bellato CM, Bedi G, Cury JA. 2006. The role of sucrose in cariogenic dental biofilm formation—new insight. J Dent Res 85:878–887. doi:10.1177/154405910608501002 16998125 PMC2257872

[6] Abranches J, Zeng L, Kajfasz JK, Palmer SR, Chakraborty B, Wen ZT, Richards VP, Brady LJ, Lemos JA. 2018. Biology of oral streptococci. Microbiol Spectr 6. doi:10.1128/microbiolspec.gpp3-0042-2018 PMC6287261 30338752

[7] Huang P, Hu P, Zhou SY, Li Q, Chen WM. 2014. Morin inhibits sortase A and subsequent biofilm formation in Streptococcus mutans. Curr Microbiol 68:47–52. doi:10.1007/s00284-013-0439-x 23982199

[8] Rudin L, Roth N, Kneubühler J, Dubey BN, Bornstein MM, Shyp V. 2023. Inhibitory effect of natural flavone luteolin on Streptococcus mutans biofilm formation. Microbiol Spectr 11:e0522322. doi:10.1128/spectrum.05223-22 37732737 PMC10581090

[9] Adil M, Singh K, Verma PK, Khan AU. 2014. Eugenol-induced suppression of biofilm-forming genes in Streptococcus mutans: an approach to inhibit biofilms. J Glob Antimicrob Resist 2:286–292. doi:10.1016/j.jgar.2014.05.006 27873689

[10] Su X, Yu H, Wang X, Zhang C, Wang H, Kong X, Qu Y, Luan Y, Meng Y, Guan J, Song G, Wang L, Song W, Zhao Y. 2022. Cyanidin chloride protects mice from methicillin-resistant Staphylococcus aureus-induced pneumonia by targeting Sortase A. Virulence 13:1434–1445. doi:10.1080/21505594.2022.2112831 35983964 PMC9397467

[11] Gopu V, Shetty PH. 2016. Cyanidin inhibits quorum signalling pathway of a food borne opportunistic pathogen. J Food Sci Technol 53:968–976. doi:10.1007/s13197-015-2031-9 27162376 PMC4837728

[12] Duarte S, Gregoire S, Singh AP, Vorsa N, Schaich K, Bowen WH, Koo H. 2006. Inhibitory effects of cranberry polyphenols on formation and acidogenicity of Streptococcus mutans biofilms. FEMS Microbiol Lett 257:50–56. doi:10.1111/j.1574-6968.2006.00147.x 16553831

[13] Bowen WH, Koo H. 2011. Biology of Streptococcus mutans-derived glucosyltransferases: role in extracellular matrix formation of cariogenic biofilms. Caries Res 45:69–86. doi:10.1159/000324598 PMC3068567 21346355

[14] Xu X, Zhou XD, Wu CD. 2012. Tea catechin epigallocatechin gallate inhibits Streptococcus mutans biofilm formation by suppressing gtf genes. Arch Oral Biol 57:678–683. doi:10.1016/j.archoralbio.2011.10.021 22169220

[15] Koo H, Nino de Guzman P, Schobel BD, Vacca Smith AV, Bowen WH. 2006. Influence of cranberry juice on glucan-mediated processes involved in Streptococcus mutans biofilm development. Caries Res 40:20–27. doi:10.1159/000088901 16352876

[16] Xu X, Zhou XD, Wu CD. 2011. The tea catechin epigallocatechin gallate suppresses cariogenic virulence factors of Streptococcus mutans. Antimicrob Agents Chemother 55:1229–1236. doi:10.1128/AAC.01016-10 21149622 PMC3067078

[17] Veloz JJ, Saavedra N, Alvear M, Zambrano T, Barrientos L, Salazar LA. 2016. Polyphenol-rich extract from propolis reduces the expression and activity of Streptococcus mutans glucosyltransferases at subinhibitory concentrations. Biomed Res Int 2016:4302706. doi:10.1155/2016/4302706 27110563 PMC4821976

[18] Koo H, Pearson SK, Scott-Anne K, Abranches J, Cury JA, Rosalen PL, Park YK, Marquis RE, Bowen WH. 2002. Effects of apigenin and tt-farnesol on glucosyltransferase activity, biofilm viability and caries development in rats. Oral Microbiol Immunol 17:337–343. doi:10.1034/j.1399-302x.2002.170602.x 12485324

[19] Zhang Q, Ma Q, Wang Y, Wu H, Zou J. 2021. Molecular mechanisms of inhibiting glucosyltransferases for biofilm formation in Streptococcus mutans. Int J Oral Sci 13:30. doi:10.1038/s41368-021-00137-1 34588414 PMC8481554

[20] Shamsudin NF, Ahmed QU, Mahmood S, Ali Shah SA, Khatib A, Mukhtar S, Alsharif MA, Parveen H, Zakaria ZA. 2022. Antibacterial effects of flavonoids and their structure-activity relationship study: a comparative interpretation. Molecules 27:1149. doi:10.3390/molecules27041149 35208939 PMC8879123

[21] Koo H, Duarte S, Murata RM, Scott-Anne K, Gregoire S, Watson GE, Singh AP, Vorsa N. 2010. Influence of cranberry proanthocyanidins on formation of biofilms by Streptococcus mutans on saliva-coated apatitic surface and on dental caries development in vivo. Caries Res 44:116–126. doi:10.1159/000296306 20234135 PMC2883842

